# Neuroendocrine neoplasms in the breast oncology field: dilemmas of nature and morphology

**DOI:** 10.3389/fendo.2023.1216424

**Published:** 2023-11-01

**Authors:** Tomonori Kawasaki, Tomoaki Tashima, Atsushi Enomoto, Jiro Ichikawa, Hirokazu Nagai, Chisako Muramatsu, Yasuhiro Nakamura, Kyoichi Kaira

**Affiliations:** ^1^ Department of Pathology, Saitama Medical University International Medical Center, Hidaka, Japan; ^2^ Comprehensive Cancer Center, Saitama Medical University International Medical Center, Hidaka, Japan; ^3^ Department of Pathology, Nagoya University Graduate School of Medicine, Aichi, Japan; ^4^ Graduate School of Medicine, University of Yamanashi, Chuo, Japan; ^5^ Clinical Research Center, Nagoya Medical Center, Aichi, Japan; ^6^ Faculty of Data Science, Shiga University, Hikone, Japan

**Keywords:** breast, diagnosis, immunohistochemistry, insulinoma-associated protein 1, neuroendocrine neoplasm, neuroendocrine tumor, treatment

## 1 Introduction

We read with great interest the article entitled ‘The Complex Histopathological and Immunohistochemical Spectrum of Neuroendocrine Tumors—An Overview of the Latest Classifications’ published in the *International Journal of Molecular Sciences* (1). Gheorghişan-Gălăţeanu et al. were concerned that over the years, the classification of neuroendocrine neoplasms (NENs) has changed significantly, often causing confusion due to clinical, molecular, and immunohistochemical variability. Therefore, a comprehensive understanding of the pivotal histological and immunohistochemical characteristics of NENs as well as an outline of the updated NEN classifications which does not rely on their site of origin could be expected to pave the way to validating possible diagnostic and prognostic markers and also guide the selection and administration of therapy (1).

## 2 Subsections relevant for the subject and discussion

In the breast oncology field, the assertion of NENs, as a cross-disciplinary disease concept in the WHO classification (5th ed.) updated in 2019, has given rise to various forms of confusion regarding diagnostic criteria as well as treatment on a global scale including in Japan (2, 3). Specifically, the existence of an authentic mammary NEN is still controversial and widely debated in the literature, with the exception of the fairly uncommon primary small cell neuroendocrine carcinoma (4–7). Since neuroendocrine cells do not essentially exist in normal lacteal glands (8), breast neuroendocrine tumor (NET) can be regarded as being embryologically different from NETs arising within endocrine organs and/or derived from pre-existing neuroendocrine cells (9, 10). In fact, mammary carcinomas with neuroendocrine features are, intriguingly, of the luminal subtype, i.e., they express estrogen and/or progesterone receptors, and rarely have a clear ‘rosette’ architecture or nuclear, ‘salt-and-pepper’ chromatin pattern. Incidentally, we are aware that NENs of gynecological origin as well as those in the head and neck regions are closely associated with specific human papillomaviruses (11, 12).

Solid papillary carcinoma (SPC) of the breast, representing primarily an *in situ* nature, is characterized by a solid growth pattern with a delicate vascular/fibrovascular network, and in our experience as well as according to the literature, almost all were immunohistochemically demonstrated to have distinct neuroendocrine features (2, 13–16). The proliferative constructions show strikingly characteristic NET morphologies, in conjunction with finely granular cytoplasm and “plasmacytoid” cell appearances. Interestingly, in neoplasms of this type, bland-appearing neuroendocrine cells and/or “neuroendocrine cell hyperplasia” can often be found in the background breast tissues (10, 17). Accordingly, among mammary cancers, SPC could be the closest, not only morphologically but also developmentally, to conventional NETs. On the other hand, particularly under invasive breast tumor conditions, we occasionally experience “carcinomas with neuroendocrine morphologies”, in which neuroendocrine differentiation is suggested, but it is not possible to determine whether the tumor has an intrinsic neuroendocrine nature. Insulinoma-associated protein 1, a new next-generation marker, has the potential to allow tumors such as NENs or neuroendocrine phenotype cancers to be identified (18–21).

Regarding immunohistochemical features of breast NENs, most cases are positive for somatostatin receptor 2 as well as the estrogen receptor, and E-cadherin, while being negative for HER1 (EGFR), HER2, occasionally with gene analysis, and high-molecular-weight cytokeratins (CKs), such as CK5/6 and CK14 (9, 22–24). Androgen receptor is also occasionally identified in NEN cells (2). The Ki67 (MIB-1) labeling index is not high in most cases (22, 23), the exception being those with neuroendocrine carcinoma (4, 6). Furthermore, CD56 (NCAM) can be expressed, but the interpretation of staining may be challenging (2). In fact, we recently demonstrated that, in the field of breast oncology, NCAM is considerably less sensitive and less specific than other neuroendocrine markers, because its antibody expression is common in normal mammary ducts and lobules without intrinsic neuroendocrine cells (25).

The possibility of metastatic NEN, including small cell carcinoma, from another site should be ruled out (4). First, it is necessary to confirm that there are no lesions raising suspicion of NEN in other organs, especially in the lung and gastrointestinal tract, on imaging and/or clinical history (2). Furthermore, the presence of an associated *in situ* lesion, or conventional-type mammary carcinoma component, confirms the primary nature of the tumor (4, 22, 26). GATA3, GCDFP-15 and/or mammaglobin immuno-expressions support a diagnosis of breast primary NEN (2). In addition, mammary NENs are generally CK7-positive and CK20-negative, whereas pulmonary small cell carcinoma is negative for both. TTF-1 expression in small cell mammary carcinoma has been reported in approximately 20% of cases (4, 27), but not diffuse strong nuclear staining as is frequently seen in small cell carcinoma of lung origin (2).

## 3 Conclusions

Overall, application of the interdisciplinary NEN concept, despite affecting a non-neuroendocrine organ, given the background of compatibility or exclusiveness of the histopathological classification based on the neuroendocrine “nature” and “morphology” of the tumors, e.g., SPC and hypercellular mucinous carcinoma, may well lead to an increasingly confusing situation in the field of breast oncology. From the perspective of molecular biology, it was recently demonstrated that breast NENs are characterized by an enrichment of mutations in transcription factors and likely constitute a spectrum of entities genomically as well as histologically related to mucinous carcinoma (28, 29). Thus, in our view, it would be better to group breast carcinomas with neuroendocrine features together as a relatively broad-based tumor entity, thereby focusing on neuroendocrine differentiation, perhaps providing a fruitful avenue to searching for novel future treatment methods.

## Author contributions

Conceptualization: TK, TT, and KK. Methodology: TK, JI, and CM. Resources: TK. Writing – Original Draft Preparation: TK, and TT. Writing – Review and Editing: AE, YN, and KK. Supervision: HN. Funding Acquisition: TK. All authors contributed significantly to the article and approved the submitted version.
